# Attentional development is altered in toddlers with congenital heart disease

**DOI:** 10.1002/jcv2.12232

**Published:** 2024-04-21

**Authors:** Alexandra F. Bonthrone, Vanessa Kyriakopoulou, Luke Mason, Andrew Chew, Shona Falconer, Christopher J. Kelly, John Simpson, Kuberan Pushparajah, Mark H. Johnson, A. David Edwards, Chiara Nosarti, Emily J. H. Jones, Serena J. Counsell

**Affiliations:** ^1^ Centre for the Developing Brain School of Biomedical Engineering and Imaging Sciences King's College London London UK; ^2^ Department of Forensic and Neurodevelopmental Sciences Institute of Psychiatry Psychology & Neuroscience King's College London London UK; ^3^ Paediatric Cardiology Department Evelina London Children's Healthcare London UK; ^4^ Department of Psychology University of Cambridge Cambridge UK; ^5^ Department of Psychological Sciences Centre for Brain and Cognitive Development Birkbeck University of London London UK; ^6^ Department of Child and Adolescent Psychiatry Institute of Psychiatry Psychology and Neuroscience King's College London London UK

**Keywords:** congenital heart disease, executive functions, eye‐tracking, visual attention

## Abstract

**Background:**

Congenital Heart Disease (CHD) is the most common congenital abnormality. Survival rates are over 90%, however infants with CHD remain at high risk of attention and executive function impairments. These abilities are difficult to assess in toddlers because clinical assessments rely on language abilities which are commonly delayed in CHD. Our aim was to characterise visual attention in toddlers with CHD compared to controls and identify associations with parent‐rated effortful control.

**Methods:**

Thirty toddlers with CHD (19 male, median (IQR) age at assessment 22.2 (22–23.1) months) and 66 controls from the developing human connectome project (36 male, age at assessment 22 (21.5–23.8) months) using eye‐tracking tasks designed to assess multiple components of visual attention. Analyses of co‐variance and regressions were used to identify differences between groups and relationships between gaze behaviours and parent‐rated effortful control.

**Results:**

Toddlers with CHD were less accurate when switching behaviours (set‐shifting) [median (IQR) 79%, (28–100)] compared to controls [100% (86–100), pFDR = 0.032], with worse accuracy associated with lower parent‐rated effortful control in CHD but not controls (interaction pFDR = 0.028). Reaction times were slower during selective [CHD 1243 ms (986–1786), controls 1065 ms (0851–1397), pFDR<0.001] and exogenous attention tasks [CHD 312 ms (279–358), control 289 (249–331), (pFDR = 0.032) and endogenous attention was less mature (prolonged looks at facial stimuli CHD 670 ms (518–885), control 500 ms (250–625), (pFDR = 0.006). These results were unrelated to differences in cognition or socioeconomic status. In contrast, the allocation of attentional resources was preserved in CHD.

**Conclusions:**

We identified a profile of altered attention and early executive functioning development in CHD. Eye‐tracking may provide clinically feasible, early objective measures of attention and executive function development in CHD.

**Key points**

- Children with Congenital Heart Disease (CHD) are at increased risk of attention and executive function impairments
- Early indicators of altered neurodevelopment may improve prognostic sensitivity, aid parental counselling and provide markers to test efficacy of potential cognitive and behavioural interventions, however attention and early executive functions are hard to assess in toddlers as they rely on language processing
- Using objective measures from eye‐tracking, we identified a profile of altered attentional control and set shifting which was unrelated to socioeconomics or general cognition
- Altered set‐shifting was associated with parent‐rated antecedents of executive functions, suggesting this may predict executive functions in childhood.
- These metrics may represent early objective measures of altered development of attention and executive functions in CHD.

## INTRODUCTION

Congenital heart disease (CHD) is the most common congenital abnormality, affecting approximately 1% of neonates (EUROCAT, 2022). While advances in clinical care have led to survival rates of over 90% (Wren & Sullivan, 2001), survivors remain at high risk of neurodevelopmental impairments (Feldmann et al., 2021; Gaynor et al., 2015) which can persist into adolescence (Bellinger et al., 2011) and adulthood (Klouda et al., 2017) and impact on quality of life (Dewey & Volkovinskaia, 2018) and educational achievement (Lawley et al., 2019). Identifying early indicators of impaired neurodevelopment in CHD may improve prognostic sensitivity, aid parental counselling and provide markers to test efficacy of potential cognitive and behavioural interventions.

Executive Functions (EF) are among the strongest predictors of quality of life in children and adolescents with CHD (Calderon & Bellinger, 2015; Jackson et al., 2021), and toddlers with CHD are at high risk of developing executive function impairments (Feldmann et al., 2021; Jackson et al., 2021). EF encompass a set of cognitive and self‐regulatory processes which support goal‐directed behaviour (Diamond, 2013). Antecedents of EF in infancy include visual attentional control, information processing and set‐shifting abilities (Hendry et al., 2016). These support emergent EF, termed ‘effortful control’, which enable deliberate control of behaviour (Anderson, 2002; Diamond, 2013). Characterizing visual attention and set‐shifting in early childhood is crucial to understanding how EF emerge, however there is little research on this in CHD.

EF assessments employed in older children are unsuitable for toddlers as they rely on non‐executive skills such as language (Hughes & Graham, 2002). Eye‐tracking is a widely used non‐invasive technology that characterizes gaze behaviours with high temporal and spatial resolutions to provide direct and objective measures (Karatekin, 2007). Attentional control and set‐shifting can be detected in toddlers using passive and gaze‐contingent eye‐tracking tasks (Braithwaite et al., 2023) and may predict later EF (Blankenship et al., 2019; Cuevas & Bell, 2014; Veer et al., 2017). Importantly, eye‐tracking tasks are not dependent on language and therefore well‐suited to measuring abilities in toddlers (Karatekin, 2007; Richmond & Nelson, 2009) including those with CHD who are at increased risk of language delays. Eye‐tracking has been used to investigate attentional processes in toddlers at risk of autism spectrum disorder/condition (ASD) and attention deficit/hyperactivity disorder (ADHD) (Gui et al., 2020; Hendry et al., 2018; Jones et al., 2017). In infants with CHD, gaze‐following behaviour at age 6 and 12 months has been assessed with eye‐tracking (Feldmann et al., 2023). At age 6 months infants with CHD and controls were not different, but at age 12 months gaze‐directed fixations were less frequent and shorter in CHD, perhaps reflecting an altered understanding of social stimuli as an attentional cue (Braithwaite et al., 2020). However, to our knowledge no studies have comprehensively assessed multiple components of visual attention in toddlers with CHD.

Colombo (2001) suggests three distinct components of visual attention emerge across infancy: alertness to exogenous stimuli (in first few months of life); followed by exogenous attentional orienting (“where”) and feature attention (“what”) (by age 6 months); finally endogenous attention, the ability to internally direct and maintain attention, (into second year of life). Others distinguish between sustained attention, the ability to maintain attention (emerges from alerting); selective attention, the ability to attend certain visual inputs while ignoring others (emerges from orienting and shifting); and executive attention, the ability to select, switch and inhibit attentional behaviours (emerges in later infancy) (Hendry et al., 2019; Johnson et al., 1991). Interestingly, these models do not account for how infants allocate attentional resources, despite evidence that infants preferentially attend information which are neither too complex to be understood nor too simple (Kidd et al., 2012).

**FIGURE 1:**
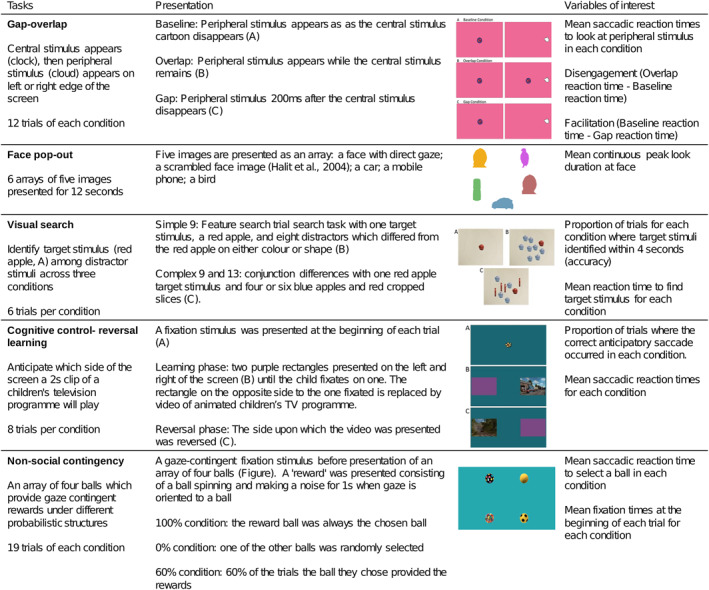
Overview of eye‐tracking task battery (adapted from Braithwaite et al. (2023).

In this study we compared antecedents of EF in toddlers with CHD to typically developing controls using eye‐tracking tasks designed to measure components of visual attention: exogenous, endogenous, and selective attention, set‐shifting and allocation of attentional resources. An overview of the eye‐tracking tasks and variables of interest is provided in Figure 1. We aimed to:

1. Assess differences in visual attention between toddlers with CHD and typically developing children from the Developing Human Connectome Project (dHCP, https://www.developingconnectome.org/). We made no prediction regarding the relationship between allocation of attentional resources and CHD given the lack of literature regarding a relationship with EF. We hypothesized that when compared to controls, children with CHD would show:
  - Slower reaction times on exogenous and selective attention tasks
  - Less mature endogenous attention (prolonged looks at facial stimuli)
  - Less accurate set‐shifting
2. Characterize the association between gaze behaviours and parent‐reated effortful control, an emergent form of EF, in toddlers with CHD and controls. We hypothesized that parent‐rated effortful control would be associated with eye‐tracking metrics identified in Aim 1 in toddlers with CHD, who are at high risk of impaired EF, but not controls.

## METHODS

### Ethical approval

The National Research Ethics Service West London committee provided ethical approval (CHD 07/H0707/105; dHCP 14/LO/1169). All toddlers who participated in this study were enrolled in the congenital heart disease imaging programme (CHD) or the developing human connectome project (controls) as fetuses or neonates. Informed written consent was obtained from parents at enrolment as fetuses or neonates, and prior to assessment.

### Recruitment

A prospective cohort of 52 toddlers with CHD recruited at birth from St Thomas' Hospital London as part of a large study investigating brain development in CHD (Bonthrone et al., 2021; Kelly et al., 2017, 2019) were eligible for an eye‐tracking assessment at age 22‐months from September 2018‐July 2021. Inclusion criteria were a diagnosis of critical or serious CHD, defined as CHD which requires intervention by catheterization or surgery before age 1 year (Ewer et al., 2011; Kelly et al., 2017). Exclusion criteria included suspected or confirmed chromosomal or syndromic disorder, neonatal surgery before recruitment (excluding cardiac catheterization) and suspected congenital infection. 33 (63%) infants attended an eye‐tracking assessment: 2 infants died, 13 declined to visit the hospital and 4 did not respond. Three infants did not comply with the eye‐tracking assessment, resulting in 30 toddlers with CHD who underwent an eye‐tracking assessment.

66 healthy toddlers from the developing Human Connectome Project (dHCP) assessed contemporaneously using the same equipment served as a control group (Edwards et al., 2022). The dHCP is a large longitudinal open‐source research programme in which participants underwent a fetal and/or neonatal MRI scan and a follow‐up assessment, including eye‐tracking, as close to 18 months corrected age as possible. Appointments were scheduled in accordance with families' availability between 2015 and 2021. 853 toddlers from the developing human connectome project met the following inclusion criteria for typically developing controls within the study period: gestational age at birth ≥34.0 weeks, no admission to the neonatal unit except for observation if born 34–37 weeks, no known congenital defects or elevated risk for ASD/ADHD, no severe brain injury on neonatal MRI. Of 853 toddlers, 511 (60%) attended a follow‐up assessment. Of 511 toddlers who attended a dHCP follow‐up assessment, 439 were assessed at <21.0 months corrected age and excluded from further analysis. Of 73 toddlers assessed at ≥21.0 months corrected age, toddlers were excluded if they had: GMFCS >0 (*n* = 3), Bayley Scales of Infant and Toddler Development‐3rd edition motor or cognitive composite score <70 (*n* = 1) and non‐compliance with eye‐tracking assessment (*n* = 3).

### Eye‐tracking assessment

Eye‐tracking was performed at 21–37 months corrected age. Data acquisition and processing matches that reported by Braithwaite et al. (2023). Detailed task presentation methods and criteria for trial validity are provided in Appendix S1.

Data were acquired from a Tobii TX‐300 (Tobii AB, Sweden) at a sampling rate of 120 Hz, with children sitting approximately 60 cm from the “23″ screen (58.42 cm × 28.6 cm, 52.0° × 26.8°, native resolution of 1920 × 1080 pixels, 16:9 aspect ratio). Stimuli were presented on an Apple (Apple Inc.) Macbook Pro, using a custom‐written stimulus presentation framework (Task Engine, sites.google.com/site/taskenginedoc/), running in Matlab using Psychtoolbox 3 (Brainard, 1997; Kleiner et al., 2007) and the GStreamer library (gstreamer.freedesktop.org) for video decoding. Five‐point calibration was performed before task administration. Post‐hoc calibration stimuli were interspersed between tasks to measure accuracy (the spatial displacement of recorded gaze from the point fixated) and precision (variability in consecutive samples on the same fixation point) throughout the session. Trial validity was calculated for each task using cutoffs for accuracy, precision and missing data samples.

The assessment started with the assessor positioning each participant in front of the eye tracker. An automatic five‐point calibration was performed. Initiation of trials for each task except for the Gap‐overlap was contingent on the participant fixating on a central fixation stimulus, at a size of 3 cm × 3 cm (2.86° × 2.86° at 60 cm viewing distance). Task administration was intermixed and distributed throughout the battery.

*Exogenous attention* was measured with the Gap‐overlap paradigm, which assesses the ability to shift fixation from a central to peripheral target when both are present (‘overlap’), and when the peripheral target appears as (‘baseline’) or after (‘gap’) the central stimulus (Johnson et al., 1991).

*Endogenous attention* was assessed with the face pop‐out paradigm, where static images of faces and objects are presented (Gliga et al., 2009; Halit et al., 2004). Infants preferentially attend facial stimuli, however unbroken gaze duration to faces decreases in the first year as the ability to endogenously direct attention develops, and infants choose to attend different objects in their environment (Hendry et al., 2018).

*Selective attention* was assessed with a visual search task (Donnelly et al., 2007; Hendry et al., 2019; Woods et al., 2013) including feature distractor stimuli, which homogeneously differ from the target, and more complex conjunction distractor stimuli, which differ from the target heterogeneously. Higher numbers of heterogenous distractor stimuli place larger demands on attention abilities. This task included three conditions: 8 feature distractors (most simple), 8 conjunction distractors, and 12 conjunction distractors (most complex).

*Set‐shifting*, a form of executive attention encompassing the ability to alter behaviour based on changes in exogenous ‘rules’ (Blakey et al., 2016), was assessed with the cognitive control‐ reversal learning paradigm (Wass et al., 2011). Anticipatory saccades were measured as a set of short videos are presented (i) on one side of a screen (learning condition) (ii) after presentation shifts to the other side (set‐shifting condition).

*The allocation of attentional resources* was assessed with a non‐social reward contingency task (Braithwaite et al., 2023) in which a set of stimuli provide gaze‐contingent rewards under different probabilistic structures: completely predicable (whichever stimulus the child looks at provides the reward), moderately predictable (60% of rewards given are provided by the ‘chosen’ stimulus) or random (the reward is never provided by the ‘chosen’ stimulus). Moderately predictable conditions are expected to elicit faster reaction times as toddlers allocate more attentional resources to the condition that is neither too simple (completely predictable) nor too complex (random) (Kidd et al., 2012).

### Neurodevelopmental assessment

During the same visit as the eye‐tracking assessment, a developmental paediatrician or paediatric psychologist administered the Bayley Scales of Infant and Toddler Development‐Third Edition(Bayley, 2006) to obtain cognitive composite and motor composite scores. Assessors also administered an age independent neurological examination (Haataja et al., 1999) and assigned a gross motor function classification from 0 (no gross neurological motor impairment) to 5 (requires a wheelchair in all settings, with impaired head and trunk posture and limb movements) (Palisano et al., 1997).

Parents completed the Early Childhood Behaviour Questionnaire‐very short form (Putnam & Rothbart, 2006). The effortful control subscale (mean of attentional control, attention focusing, low intensity pleasure and perceptual sensitivity items) was calculated (higher scores represent better effortful control). One toddler with CHD did not complete the neurodevelopmental assessment.

Travel and sustenance were reimbursed for all participants and their families.

### Socioeconomic status

Index of multiple deprivation, a composite measure of socioeconomic status in England encompassing factors related to income, employment, education, health, and crime (https://imd‐by‐postcode.opendatacommunities.org/), was calculated from maternal postcode at birth using the 2015 data release and reported as quintiles.

### Statistical analysis

Analyses were performed in R v3.6.2. Normality was tested using Shapiro‐Wilk and skewness/kurtosis. Differences in demographics, neurodevelopment and data quality between groups were assessed with Mann‐Witney U, Kruskal‐wallis, *t*‐tests, fisher's exact and *χ* ^2^.

Repeated measures Analysis of Covariance (ANCOVA) assessed differences between groups for each eye‐tracking task (within‐participant factor: task condition; between‐participant factor: CHD). In keeping with the literature (Latal, 2016; Sanapo et al., 2016), cognitive composite scores and gestational age at birth were significantly different between groups and included as covariates in all models. Sex, socioeconomic status, eye‐tracking data quality (accuracy and precision) and corrected age at assessment were assessed for inclusion as covariates using spearman's rank correlations, mann‐whitney U and kruskal‐wallis (variables with *p* < 0.1 were included; see Table S1). Main effects of CHD and CHD*task condition interactions were assessed for seven eye‐tracking measures: gap‐overlap reaction times; visual search proportion of correct trials and reaction times; cognitive control‐reversal learning proportion of correct saccades and reaction times; non‐social contingency reaction times to picking a ball and to fixation stimulus. The effect of CHD on peak face look durations in the face pop‐out was assessed with ANCOVA. Effect sizes (ηp^2^, 0.01–0.06 small effect, 0.061–0.14 medium effect size, >0.14 large effect) were reported. *p*‐values were adjusted for 15 comparisons using the False Discovery Rate (FDR) (Benjamini & Hochberg, 1995). F‐values/regression coefficients and uncorrected *p*‐values for covariates in all models are reported in Tables S3.

Multiple linear regressions were used to investigate whether CHD*parent‐rated effortful control interactions predicted eye‐tracking metrics identified in the ANCOVA analysis. The relationship between parent‐rated effortful control scores and sex, socioeconomic status and corrected age at assessment was assessed (Table S2) however model covariates were the same as ANCOVAs. Proportion of correct anticipatory saccades on cognitive control‐ reversal learning was highly skewed. Therefore, it was converted to number of correct saccades and entered into a poisson regression with log(number of valid trials) included as an offset.

Linearity, homoscedasticity, independence and normality were checked with GVLMA. Where assumptions for multiple regression were not met, robust regression (lmrob from robustbase) was performed (Lourenço et al., 2011; Maechler et al., 2021). Unstandardized coefficients (B), standard errors and *p*‐values for interaction terms are reported. *p*‐values were adjusted for seven comparisons. F‐values/regression coefficients and uncorrected *p*‐values for covariates in all models are reported in Tables S4. Finally, Post‐hoc regressions were run with CHD as a covariate to identify relationships between parent‐rated effortful control and eye‐tracking metrics across the whole sample.

## RESULTS

Demographic characteristics are summarized in Table 1. Toddlers with CHD were born at a younger gestational age and had lower cognitive and motor composite scores than age‐matched controls. There were no additional significant demographic differences between groups.

**TABLE 1:** Demographic information in toddlers with CHD and control children.

	CHD (*N* = 30)	Control (*N* = 66)	Difference between groups
Gestational age at birth in weeks, median (IQR)	38.71 (38.32–39.00)	40 (39–40.71)	**U = 1402, *p* = 0.001**
CHD type			
Defects causing abnormal streaming of blood			
Transposition of the great arteries, *n* (%)	13 (43)	‐	‐
Left‐sided heart defects			
Coarctation of the Aorta, *n* (%)	9 (30)	‐	‐
Aortic stenosis with coarctation of the aorta, *n* (%)	1 (3)	‐	‐
Right‐sided heart defects			
Pulmonary Stenosis	3 (10)	‐	‐
Pulmonary Atresia	2 (7)	‐	‐
Tetralogy of Fallot	2 (7)	‐	‐
Age at assessment in months corrected for gestational age at birth, median (IQR)	22.2 (22.0–23.1)	22 (21.5–23.8)	*U* = 826.5, *p* = 0.198
Male, *n* (%)	19 (63)	36 (55)	0.507
IMD Quintile, *n* (%)			
1 (most deprived)	5 (17)	13 (20)	0.856
2	6 (20)	19 (29)	
3	8 (27)	15 (23)	
4	7 (23)	11 (17)	
5	4 (13)	8 (12)	
Follow‐up assessment			
Bayley‐III			
Cognitive composite score, median (IQR)	95 (85–100)	100 (95–105)	**U = 1340, *p* = 0.002**
Motor composite score, mean (SD)	95.8 (7.6)	101.3 (9.5)	**t(63) = 3.0, *p* = 0.004**
Early childhood behaviour checklist (ECBQ)			
ECBQ parent‐rated effortful control, median (IQR)	4.58 (4.17–5.00)	4.67 (4.13–5.00)	*U* = 922.5 *p* = 0.967
	Cronbach's *α* = 0.69	Cronbach's *α* = 0.64	

*Note*: Results in bold are significant.

Abbreviations: IQR, interquartile range; SD standard deviation.

Eye‐tracking accuracy, precision, and proportion of toddlers with valid data in each task did not differ between groups (Table S5). Socioeconomic status was unrelated to gaze behaviours (Table S1).

### Participation bias in toddlers with CHD

When comparing toddlers who attended the eye‐tracking assessment (*n* = 33; 30 in analysis sample and three who attended but did not comply) and those who did not attend for reasons other than death (*n* = 17; 13 did not want to travel, 4 did not respond), there were no significant differences in index of multiple deprivation (*p* = 0.517) or birth below 37 weeks (*p* = 0.542). However, the proportion of toddlers with single ventricle physiology who did not attend the eye‐tracking assessment (*n* = 4 of 17) was significantly higher than the number who did (*n* = 1 of 33; *p* = 0.039).

Importantly, 11 toddlers for whom eye‐tracking data were not acquired within the study period underwent a neurodevelopmental assessment (5 home visit, 3 attended the hospital outside of the study period, 3 did not comply with the eye‐tracking assessment). This included all toddlers with single ventricle physiology who did not undergo eye‐tracking. There were no significant differences in cognitive [median (IQR) 100 (93–100), *p* = 0.413] and motor [94 (94–102), *p* = 0.648] composite scores between toddlers who underwent a neurodevelopmental assessment but not eye‐tracking (*n* = 11) and infants who were included in the analysis sample (*n* = 30).

### Participation bias in typically developing toddlers

There were no significant differences in socioeconomic status between the toddlers who did not attend follow‐up (*n* = 342), attended follow‐up at <21.0 months corrected age (*n* = 348), and attended follow‐up at ≥21.0 months corrected age (*n* = 73; *χ* ^2^ = 7.7186, *p* = 0.461)

### Differences between toddlers with CHD and controls

Results are summarized in Table 2.

**TABLE 2:** Differences in eye‐tracking metrics between toddlers with CHD and controls.

	CHD median (IQR)	Control median (IQR)	Overall effect of CHD	Interaction between CHD and condition
Gap‐overlap				
Reaction times (milliseconds)	Baseline: 327 (297–345)	Baseline: 291 (277–316) Gap: 242 (227–255)	**F(1, 75) = 6.97 p**_**FDR**_ ** = 0.032 ηp** ^**2**^ ** = 0.09**	F(2, 160) = 0.192 p_FDR _= 0.825 ηp^2^<0.001
	Gap: 255 (243–276) Overlap: 363 (316–427)	Overlap: 336 (308–390)		
Face pop‐out				
Continuous look durations at faces (milliseconds)	670 (518–875)	500 (250–625)	**F(1,65) = 12.22 p**_**FDR**_ ** = 0.006 ηp^2^ = 0.16,**	‐
Visual search				
Visual search proportion of correct trials	Feature 9: 0.83 (0.67–1.0)	Feature 9: 0.83 (0.67–1.0)	F(1,81) = 3.44 p_FDR_ = 0.112 ηp^2^ = 0.04	F(2,172) = 3.93 p_FDR_ = 0.053 ηp^2^ = 0.04
	Conjunction 9: 0.42 (0.33–0.57)	Conjunction 9: 0.50 (0.33–0.67)		
	Conjunction 13: 0.33 (0.31–0.50)	Conjunction 13: 0.50 (0.33–0.67)		
Visual search reaction times (milliseconds)	Feature 9: 904 (801–1126)	Feature 9: 851 (683–1032) conjunction 9: 1158 (980–1437) conjunction 13: 1355 (1018–1552)	**F(1,79) = 18.82 p**_**FDR**_ **<0.001 ηp** ^**2**^ ** = 0.19**	**F(2,169) = 4.67 p**_**FDR = **_ **0.032 ηp** ^**2**^ ** = 0.05**
	Conjunction 9: 1462 (1142–1806)			
	Conjunction 13: 1751 (1250–1986)			
Cognitive control‐ reversal learning				
Proportion of correct saccades	Learning: 0.95, (0.83–1)	Learning: 0.83 (0.75–1)	F(1,51) = 4.68 p_FDR_ = 0.076 ηp^2^ = 0.08	**F(1,54) = 7.28 p**_**FDR**_ = **0.032 ηp** ^**2**^ ** = 0.12**
	Reversal: 0.79, (0.28–1)	Reversal: 1 (0.86–1)		
Reaction times (milliseconds)	Learning: 681 (527–765)	Learning: 635 (575–786)	F(1,52) = 0.296 p_FDR_ = 0.663 ηp^2^ = 0.006	F(1,54) = 0.382 p_FDR_ = 0.663 ηp^2^ = 0.008
	Reversal: 613 (514–750)	Reversal: 635 (491–747)		
Non‐social contingency				
Reaction times to pick a ball (milliseconds)	0%: 525 (481–565)	0%: 493 (461–534)	F(1,52) = 0.745 p_FDR_ = 0.535 ηp^2^ = 0.01	F(2,112) = 2.96 p_FDR_ = 0.105 ηp^2^ = 0.02
	60%: 527 (501–575)	60%: 520 (491–541)		
	100%: 523 (467–556)	100%: 528 (506–569)		
Reaction times to fixation (milliseconds)	0%: 465 (407–598)	0%: 429 (366–520)	F(1,50) = 0.251 p_FDR_ = 0.663 ηp^2^ = 0.004	F(2,112) = 2.58 p_FDR_ = 0.122 ηp^2^ = 0.04
	60%: 440 (364–510)	60%: 422 (374–520)		
	100%: 652 (456–803)	100%: 555 (412–675)		

*Note*: Results in bold are significant.

#### Gap‐overlap (exogenous attention)

**FIGURE 2:**
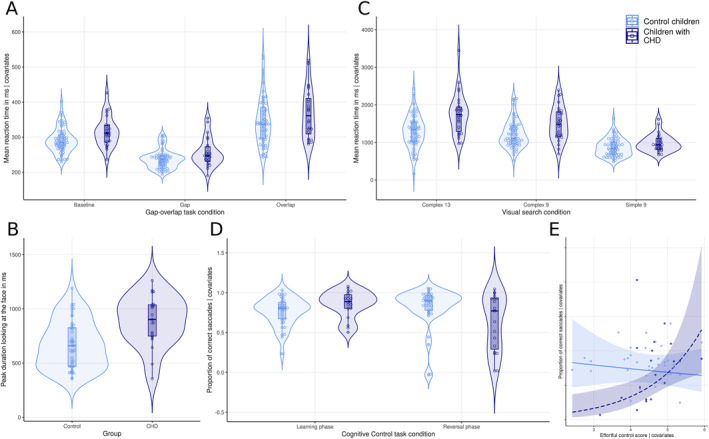
Partial plots showing differences between toddlers with CHD (dark) and controls (light) in the (A) Cognitive control‐ reversal learning (B) visual search (C) Gap‐overlap and (D) Face pop‐out tasks and (E) association between proportion of correct saccades in the reversal condition of the cognitive control‐reversal learning task and parent‐rated effortful control scores, adjusting for covariates.

There was a main effect of CHD on gap‐overlap reaction times (Figure 2A). Post‐hoc analyses revealed reaction times were slower in the baseline [F(1,75) = 9.21 *p* = 0.003 ηp^2^ = 0.11] and gap [F(1,75) = 7.91 *p* = 0.006 ηp^2^ = 0.10] but not overlap [ F(1,75) = 2.38 *p* = 0.127 ηp^2^ = 0.03] conditions. Disengagement [F(1,75) = 0.360 *p* = 0.550 ηp^2^ = 0.004) and facilitation (F(1,75) = 0.228 *p* = 0.634 ηp^2^ = 0.003] were not different.

#### Face pop‐out (endogenous attention)

Toddlers with CHD had longer continuous look durations at faces compared to controls (Figure 2B).

#### Visual search (selective attention)

There was a main effect of CHD and a CHD*condition interaction when comparing visual search reaction times (Figure 2C). Post‐hoc analyses revealed differences across all conditions, with effects sizes increasing with task complexity [Feature 9 (F(1,82) = 4.01), *p* = 0.049, ηp^2^ = 0.05; Conjunction 9 F(1,79) = 7.64 *p* = 0.007 ηp^2^ = 0.09; Conjunction 13 F(1,82) = 17.94 *p* < 0.001 ηp^2^ = 0.18].

#### Cognitive control‐ reversal learning (set‐shifting)

The CHD*condition interaction was significant when comparing proportion of correct saccades during cognitive control‐reversal learning. Post‐hoc analysis revealed that toddlers with CHD were significantly less accurate in the reversal [F(1,48) = 9.12, *p* = 0.004, ηp^2^ = 0.16] but not learning [F(1,82) = 2.33, *p* = 0.131, ηp^2^ = 0.03] condition (Figure 2D).

#### Non‐social contingency (allocation of attentional resources)

There were no significant differences in the non‐social contingency between toddlers with CHD and controls.

### Associations between eye‐tracking measures and parent‐rated effortful control

There was a significant CHD*parent‐rated effortful control interaction when predicting accuracy on the cognitive control‐ reversal learning reversal condition (Table 3; Figure 2E). Better accuracy associated with higher parent‐rated effortful control scores in CHD [B(SE) = 0.691 (0.263), *p* = 0.008] but not controls [B(SE) = −0.058 (0.123), *p* = 0.637]. There were no relationships between parent‐rated effortful control and eye‐tracking metrics across the whole sample (Table S6).

**TABLE 3:** Interactions between CHD and parent‐rated effortful control to predict eye‐tracking metrics.

Gap‐overlap baseline mean reaction time	B(SE) = 0.007 (0.050), p_FDR_ = 0.886
Gap‐overlap Gap mean reaction time^a^	B(SE) = 0.079 (0.044), p_FDR_ = 0.147
Cognitive control‐ reversal learning reversal condition proportion correct^b^	**B(SE) = 0.782 (0.272)** p_FDR_ ** = 0.028**
Visual search simple 9 mean reaction time^a^	B(SE) = −144 (88) p_FDR_ = 0.147
Visual search complex 9 mean reaction time	B(SE) = −291 (151) p_FDR_ = 0.147
Visual search complex 13 mean reaction time	B(SE) = −308 (184) p_FDR_ = 0.147
Pop‐out face peak look duration	B(SE) = −0.089 (0.110) p_FDR_ = 0.495

*Note*: Results in bold are significant.

^a^ robust regression.

^b^ poisson regression.

## DISCUSSION

In this study, we investigated visual attention in toddlers with CHD using a comprehensive eye‐tracking battery. Toddlers with CHD were less accurate on a set‐shifting task (cognitive control‐ reversal learning) and the degree of altered performance was associated with poorer parent‐reated effortful control suggesting this may be a marker for altered emergent EF. Gaze behaviours during selective (visual search) and endogenous (face pop‐out) attention tasks were also altered in CHD. Reaction times during the gap‐overlap were slower, driven by the gap and baseline conditions perhaps reflecting deficits in general information processing or rapid exogenous orienting. Finally, reaction times during the non‐social contingency were not different, suggesting that the allocation of attentional resources was preserved in CHD.

Attentional control, the ability to voluntarily direct attentional resources, develops over the first 2 years of life: attention towards exogenous stimuli develops in the first year (Johnson et al., 1991) while endogenously directed attention emerges in the second (Petersen & Posner, 2012). Typically developing infants preferentially orient to faces. Younger infants will maintain attention to faces, but as attentional control develops older infants endogenously shift their attention to explore other objects. Longer looks at the face during the face pop‐out task, as seen in toddlers with CHD, may reflect altered development of endogenous attention (Hendry et al., 2018). Interestingly, during visual search, which combines exogenous and endogenous attentional processes, reaction times were increasingly altered as the task became more complex suggesting infants with CHD had difficulty selectively filtering visual information and shifting between stimuli. Visual foraging refers to the ability to efficiently control the balance of attentional resources between seeking new information and processing information currently attended (Gliga et al., 2018). Longer peak face look durations and slower visual search reaction times may reflect a preference for allocating attention towards processing information currently attended over seeking new information. Overall, these results suggest attentional control, the ability to adjust attention to fit task demands, is altered in toddlers with CHD.

Although we identified a profile of altered attentional control in CHD this was unrelated to parent‐rated effortful control. However, these deficits partially overlap with those reported in toddlers at genetic risk of or diagnosed with ASD and ADHD (Gliga et al., 2009, 2018; Gui et al., 2020; Hendry et al., 2018; Jones et al., 2017). Toddlers with CHD are at increased risk of developing ADHD and ASD (Gonzalez et al., 2021; Sigmon et al., 2019). Performance on these attentional control tasks may represent early markers for symptoms of ASD and ADHD in this population, however further research is needed to test this hypothesis.

Reaction times during the gap‐overlap were longer in CHD, but facilitation (difference between baseline and gap reaction times) and disengagement (difference between the baseline and overlap) were not different. A previous study reported saccades towards a cued peripheral object were shorter and less frequent in twelve‐month‐olds with CHD compared to controls (Feldmann et al., 2023) which may reflect altered orienting to social stimuli. As reaction times were not measured in that study it is difficult to draw direct comparisons with our findings, however taken together this may reflect an impairment in rapid exogenous orienting. Of note, altered orienting may be more easily detectable at age 12 months, before the development of endogenous attention. Another possibility is that slower initiation of saccades reflects altered information processing (Elison et al., 2013; Kano et al., 2011). Indeed, toddlers with CHD were also slower to identify targets during visual search. Slower processing speeds have been reported in older survivors (Feldmann et al., 2021; Mills et al., 2018; Schlosser et al., 2022), however, to our knowledge no study has investigated information processing in toddlers with CHD. Altered information processing may be an early antecedent of poor EF (Anderson, 2002; Diamond, 2013) and slower information processing may alter attentional development (Hendry et al., 2016). Longitudinal studies in infants and toddlers with CHD are required to disentangle the interaction between information processing and exogenous attentional control.

During cognitive control‐reversal learning, toddlers with CHD were able to learn the first set of task ‘rules’ but were unable to efficiently inhibit the original response and shift to new one (set‐shifting). Importantly, the degree of deficit was associated with lower parent‐rated effortful control scores, an antecedent of EF (Hendry et al., 2016), suggesting accuracy on the reversal condition of the cognitive control‐task may be an early correlate of emergent EF in CHD.

Attentional control, information processing and set‐shifting are the foundations of cognitive flexibility (Hendry et al., 2016), a component of EF which facilitates rapid switching between and adjustment of behaviours and is moderately impaired in school children with CHD (Jackson et al., 2021). Overall, these results suggest eye‐tracking may provide clinically‐feasible screening tools for altered cognitive flexibility. Future studies should determine whether a single eye‐tracking measure or a composite variable representing cognitive flexibility best predicts later EF. Normative modelling approaches would allow clinicians to understand the degree of altered attention development in individual infants by mapping gaze behaviours onto performance in the typical population (Marquand et al., 2019).

It is important to note that these findings represent objective measures of visual attention and are adjusted for general cognitive abilities. EF are often assessed with parent ratings. However, studies in paediatric populations suggest that parent‐rated and objectively assessed executive function measures are not highly correlated (Gross et al., 2015; Lafavor et al., 2022; Soto et al., 2020). It is likely that parental ratings and objective measures are probing different constructs, as parents identify apparent difficulties during day‐to‐day situations while objective measures target aspects of EF in a structured environment (Gardiner et al., 2017).

There were no differences in parent‐rated effortful control between toddlers with CHD and typically developing controls. Parent ratings of cognitive and executive functions in children likely represent day‐to‐day difficulties while performance‐based measures target aspects of EF in a structured environment (Gardiner et al., 2017). A meta‐analysis of performance‐based assessments of cognitive flexibility and set‐shifting (which form part of effortful control) in individuals with CHD older than 3 years old reported moderate to severe deficits [mean difference −0.628 (−0.726–0.531)] when compared to healthy controls with a moderate amount of heterogeneity (Jackson et al., 2021). However, in parent‐ratings of executive functions, while overall moderate deficits were reported, studies were characterised by a high degree of heterogeneity (Jackson et al., 2021). In our recent paper there were no differences in parent‐rated executive functions in children with CHD aged 4‐6 when compared to typically developing controls (Chew et al., 2024). It is possible that at age 22 months the impact of altered attention and executive functions on day‐to‐day situations in toddlers with CHD are not significant enough to be detected by parents. Indeed, in Hendry and colleagues' (2018) study of toddlers and low and high risk of autism spectrum condition (ASC), peak look durations at the face during the face pop‐out eye‐tracking task were shorter in 15‐month‐old toddlers at high‐risk of ASC when compared to low‐risk controls. Yet within the high‐risk group, gaze behaviours in 15‐month‐old toddlers who later met the diagnostic criteria for ASC at 3 years did not differ from those who did not. At 3 years of age, when compared to controls, effortful control scores were lower in high‐risk toddlers who met the diagnostic criteria for ASC, but not high‐risk toddlers who did not. Overall, we hypothesise that while eye‐tracking measures detect early differences in attention and executive functions, parental ratings are not sufficiently sensitive to detect early differences in executive functions. However, further research is necessary to test this hypothesis.

## LIMITATIONS

Our study has some limitations. The sample size is relatively small, limiting the generalizability of these findings. Future studies with larger samples are required to assess the relationship between clinical and environmental factors and attentional development in CHD. In addition, future studies, when these toddlers are preparing to attend school, will assess the impact of altered early attention development on later EF.

It is important to note that the study period encompassed the Covid‐19 pandemic which was associated with high levels of stress in parents of children with CHD, particularly around hospitals and healthcare provision (Harvey, 2023). This likely lowered participation rates for research in hospital settings. Studies using portable eye‐tracking technologies may allow for large‐scale data collection outside of the hospital environment.

## CONCLUSIONS

Using a comprehensive eye‐tracking battery we have identified a profile consistent with altered attentional development in toddlers with CHD characterized by altered attentional control and set‐shifting. Altered set‐shifting was associated with parent‐rated antecedents of EF, suggesting this may predict EF in childhood. These metrics may represent early objective measures of altered development of attention and EF in CHD.

## AUTHOR CONTRIBUTIONS

**Alexandra F. Bonthrone**: Data curation; formal analysis; investigation; writing – original draft. **Vanessa Kyriakopoulou**: Data curation; investigation; writing – review & editing. **Luke Mason**: Methodology; software; validation; writing – review & editing. **Andrew Chew**: Data curation; investigation; writing – review & editing. **Shona Falconer**: Data curation; investigation; writing – review & editing. **Christopher J. Kelly**: Data curation; writing – review & editing. **John Simpson**: Supervision; Writing – review & editing. **Kuberan Pushparajah**: Supervision; writing – review & editing, **Mark H. Johnson**: Methodology; resources; validation; writing – review & editing. **A. David Edwards**: Conceptualization; data curation; funding acquisition; methodology; project administration; resources; writing – review & editing. **Chiara Nosarti**: Conceptualization; methodology; writing – review & editing. **Emily J. H. Jones**: Conceptualization; data curation; formal analysis; methodology; resources; software; supervision; writing – review & editing. **Serena J. Counsell**: Conceptualization; funding acquisition; investigation; methodology; project administration; resources; supervision; writing – review & editing.

## CONFLICT OF INTEREST STATEMENT

The authors have declared they have no competing or potential conflicts of interest.

## ETHICAL CONSIDERATIONS

The National Research Ethics Service West London committee provided ethical approval (CHD 07/H0707/105; dHCP 14/LO/1169). All toddlers who participated in this study were enrolled in the congenital heart disease imaging programme (CHD) or the developing human connectome project (controls) as fetuses or neonates. Informed written consent was obtained from parents at enrolment as fetuses or neonates, and prior to assessment.

## Supporting information

Supporting Information S1

Supporting Information S2

## Data Availability

Data from the Developing Human Connectome Project will be made available as part of a public data release. Data from children with Congenital Heart Disease is available from the corresponding author upon reasonable request.
