# Supplementary material for: Attentional development is altered in toddlers with congenital heart disease
Source: JCPP Adv. 2024 Apr 21;4(3):e12232. doi: 10.1002/jcv2.12232 (PMC11472800; doi:10.1002/jcv2.12232)
Supplement: Supplementary file 1 — Supporting Information S1 [file JCV2-4-e12232-s001.docx]

**Appendix S1**

Detailed eye-tracking task presentation and data preprocessing methods

*Gap Overlap*

The baseline condition consisted of a central stimulus (clock), followed immediately by a peripheral stimulus (cloud) an either the left or right side of the screen. In the overlap condition the clock continued to be presented while the cloud was presented. In the gap condition the clock was removed from the screen 200ms before presentation of the cloud.

Trials were presented in blocks of 12. Each trial started with the onset of the central stimulus accompanied by an alerting sound. When the infant fixated the clock, after 600-700ms the peripheral stimulus was presented at 21.7° of visual angle, 3cm (2.86°) from the edge, on the left or right of the screen (random), rotating at 500° per second until fixated and accompanied by sound. In all trials, a reward stimulus was then presented at the location of the cloud for 1000ms.

Trials were considered invalid and excluded if the infant did not gaze at both the clock and the cloud; if gaze was not on the clock at onset of the cloud, if >100ms of gaze data was missing between presentation of and gaze at cloud, if the child gazed at the opposite side of the screen to the cloud for >75ms or if reaction times were <150ms or >1200ms. A minimum of six valid trials per condition was required for further analysis.

Mean saccadic reaction times to the cloud were calculated for gap, overlap and baseline conditions from valid trials. Disengagement (Overlap reaction time - Baseline reaction time), the ability to orient attention from the central stimulus (clock) to peripheral stimulus (cloud) and Facilitation (Baseline reaction time - Gap reaction time), the ability to use the disappearance of clock as visual cue to prepare for peripheral target appearance, were also calculated.

*Face Pop-out*

Six visual arrays each composed of five objects were presented for 15 seconds: a face with direct gaze, a visual ‘noise’ image generated by randomising the phase spectra of the face whilst keeping the amplitude and colour spectra (Halit et al., 2004), a bird, a car and a mobile phone.

Trials were considered invalid and excluded if gaze data was available for <25% of stimulus presentation or if trials were presented for <5 seconds (due to skipping trials). Mean face peak look duration was taken as the longest contiguous run of gaze samples on the face in each trial averaged across valid trials.

*Visual Search*

Search trials consisted of three different items: red apple (target, 4.57cm x 4.57cm, 4.3° x 4.3°), blue apple (colour distractor, 4.57cm x 4.57cm, 4.3° x 4.3°) and a red cropped slice of an apple (an elongated rectangle, cropped from the full apple image, shape distractor, 1.12cm x 6.67cm, 1.1° x 6.4°). Feature search trials are simple search tasks with one target stimulus, a red apple, and eight distractors which differed from the red apple on either colour or shape (a red apple surrounded by blue apples, or a red apple surrounded by red cropped slices), labelled as Simple 9. More complex search tasks have conjunction differences with one red apple target stimulus and four or six blue apples and red cropped slices, labelled as complex 9 and complex 13.

Each trial began with a fixation stimulus of a red apple ‘flying’ into the centre of the screen from one edge over 800ms which faded over 750ms once the participant fixated on it. The target stimulus was then positioned at a random point within a circle (diameter 27.2cm, 25.5° @ 60cm), ensuring that it was not within 6cm (5.7° @ 60cm) of the centre of the screen, where it would overlap with the central fixation stimulus presented at the start of each trial. Each distractor stimulus was placed at a random location within the circle, ensuring that no stimulus (target or distractor) overlapped the spatial location of any other. The image was presented for 4000ms or until the participant fixated on the red apple, after which the red apple span as a visual reward.

Trials were considered invalid and excluded if reaction time to fixate on the target red apple was less than 150ms. Data was analysed from infants with at least three valid trials per condition. Accuracy was calculated as the number of trials on which the infant fixated the target stimulus within 4000ms divided by the total number of trials administered. Saccadic reaction time to find the target on each trial was the mean difference between the time at which the search slide was presented and the time of the first gaze sample at the target for each condition.

*Cognitive Control- Reversal Learning*

A fixation stimulus was presented at the beginning of each trial. In the learning condition, two purple rectangles (16.1° x 12.5° @ 60cm) were presented on the left and right of the screen (1.5cm, or 1.43° from the outermost edge) until the child fixated on one. If the child did not fixate on one of the rectangles after 2000ms, a rectangle was selected randomly. In the following trials, the rectangle on the opposite side to the one fixated by the infant was replaced by video of the same dimensions, showing a two second clip of the animated children’s TV programme Thomas the Tank Engine. The learning condition ended either after the child made three correct anticipatory saccades, i.e. anticipated the appearance of the video on the opposite side to the rectangle fixated upon, or eight trials had been presented.

The reversal condition followed the learning condition and consisted of nine trials where the side upon which the video was presented was reversed. The first trial of the reversal condition was not scored but served to indicate to the child that the presentation side had been reversed.

Trials were considered invalid if saccadic reaction times were less than 300ms or if no saccade was made. Participants were excluded from analysis if they made fewer than two valid trials per condition. Areas of interest were placed around the location of each of the rectangles (within one of which the video played) and dilated by 2° to account for poor calibration. Accuracy was calculated as number of correct saccades/ total number of trials where an anticipatory saccade occurred, and saccadic reaction times were averaged across valid trials.

*Non-social contingency*

This task consisted of three blocks of 19 trials, with each block corresponding to a different reward structure. Blocks were randomly distributed across the battery but constrained such that the 60% condition was always presented within the first two blocks.

At the start and end of each block, a static picture of four balls was presented for 5 seconds. In each trial, participants were presented with a fixation stimulus (hummingbird, 3cm x 3cm, 2.86° x 2.86°) that remained on screen until it was fixated. The fixation stimulus was replaced by a display of four balls (4.5cm, 2.3°), one in each corner of the screen 3cm (2.9°) from each edge. Participants oriented their gaze to one of the four balls (within an area of interest 50% bigger than the ball, subtending 4.3° degrees of visual angle); the ball they selected became their ‘chosen’ ball for that trial. The ‘reward’ for choosing a ball was that one of the balls would become animated for 1000ms; in the 100% reward block it was always the ball they chose that was animated; in the 0% reward block it was never the ball they chose (another was randomly selected) and in the 60% reward block, the ball they chose was animated in on 60% of trials (and on the remaining 40%, a randomly chosen ball was animated).

Trials were excluded if the reward was not played (to ensure the child had selected a ball) or if a reaction time was less than 150ms (to avoid trials where the child was looking at a ball when the trial started) or longer than 2s (to avoid trials where the child was disengaged); skipped trials were also excluded. Mean saccadic reaction time to select a ball across valid trials and Mean fixation times to return to the hummingbird at the beginning of each trial (an index of engagement) were calculated for each condition.
