# Supplementary material for: Attentional development is altered in toddlers with congenital heart disease
Source: JCPP Adv. 2024 Apr 21;4(3):e12232. doi: 10.1002/jcv2.12232 (PMC11472800; doi:10.1002/jcv2.12232)
Supplement: Supplementary file 2 — Supporting Information S2 [file JCV2-4-e12232-s002.docx]

**Supplementary Tables**

| Table S1. Potential covariates for each eye-tracking task | | | | | |
| --- | --- | --- | --- | --- | --- |
|  | Corrected Age at Assessment | Multiple Deprivation Index Quintile | Sex | precision | accuracy |
| Gap-overlap task reaction times | Baseline: ρ=-0.024 p=0.509  Gap: ρ=-0.048 p=0.662  Overlap: ρ=0.081 p=0.469 | Baseline: χ2= 5.93 p=0.205  Gap: χ2= 5.44 p=0.245  Overlap: χ2=4.631 p=0.327 | Baseline: U=680 p=0.129  Gap: U=714 p=0.228  **Overlap: U=635 p=0.053** | Baseline: ρ=0.038 p=0.734  **Gap: ρ=0.280 p=0.010**  Overlap: ρ=0.079 p=0.479 | Baseline: ρ=0.076 p=0.496  **Gap: ρ=0.215 p=0.051**  Overlap: ρ=0.050 p=0.651 |
| Non-social contingency reaction times to pick a ball | **Zero: ρ=0.223 p=0.064**  Sixty: ρ=-0.065 p=0.557  Hundred: ρ=0.101 p=0.404 | Zero: χ2=0.637 p=0.959  Sixty: χ2=5.18 p=0.269  Hundred: χ2=4.85 p=0.303 | **Zero: U=381 p=0.016**  **Sixty: U=671 p=0.079**  **Hundred: U=822 p=0.010** | Zero: ρ=0.114 p=0.358  Sixty: ρ=-0.159 p=0.166  Hundred: ρ=0.045 p=0.721 | Zero: ρ=0.024 p=0.845  Sixty: ρ=-0.040 p=0.727  Hundred: ρ=-0.054 p=0.670 |
| Non-social contingency reaction times to fixation stimulus | **Zero: ρ=-0.205 p=0.089**  Sixty: -0.042 p=0.702  Hundred: ρ=-0.010 p=0.931 | Zero: χ2=5.35 p=0.253  Sixty: χ2=1.37 p=0.849  Hundred: χ2=2.24 p=0.692 | Zero: U=593 p=0.886  Sixty: U=774 p=0.408  **Hundred: U=449 p=0.067** | Zero: ρ=0.157 p=0.205  **Sixty: ρ=0.268 p=0.019**  **Hundred: ρ=0.216 p=0.082** | Zero: ρ=0.154 p=0.212  **Sixty: ρ=0.282 p=0.013**  Hundred: ρ=0.194 p=0.118 |
| Cognitive control- reversal learning Proportion Correct | Learning: ρ=0.010 p=0.940  Reversal: ρ=-0.054 p=0.693 | Learning: χ2=2.45 p=0.654  Reversal: χ2=4.39 p=0.356 | Learning: U=328 p=0.296  **Reversal: U=300 p=0.095** | Learning: ρ=0.074 p=0.586  Reversal: ρ=-0.114 p=0.586 | Learning: ρ=0.010 p=0.938  Reversal: ρ=-0.021 p=0.874 |
| Cognitive control- reversal learning reaction times | Learning: ρ=0.188 p=0.160  Reversal: ρ=-0.070 p=0.603 | Learning: χ2=3.58 p=0.467  Reversal: χ2=5.40 p=0.249 | Learning: U=400 p=0.891  Reversal: U=367 p=0.705 | Learning: ρ=-0.006 p=0.963  Reversal: ρ=-0.189 p=0.158 | Learning: ρ=0.073 p=0.591  Reversal: ρ=-0.184 p=0.171 |
| Visual search proportion correct | Simple 9: ρ=0.090 p=0.404  Complex 9: ρ=-0.037 p=0.728  Complex 13: ρ<-0.001 p=0.997 | Simple 9: χ2=2.50 p=0.645  Complex 9: χ2=2.28 p=0.684  Complex 13: χ2=1.43 p=0.997 | Simple 9: U=858 p=0.340  Complex 9: U=903 p=0.579  **Complex 13: U=740 p=0.053** | **Simple 9: ρ=-0.213 p=0.045**  Complex 9: ρ=-0.032 p=0.766  Complex 13: ρ=-0.138 p=0.198 | **Simple 9: ρ=-0.243 p=0.022**  Complex 9: ρ=-0.126 p=0.239  Complex 13: ρ=-0.112 p=0.295 |
| Visual search reaction times | Simple 9: ρ=-0.164 p=0.124  Complex 9: ρ=-0.036 p=0.740  **Complex 13: ρ=0.178 p=0.096** | Simple 9: χ2=3.15 p=0.534  Complex 9: χ2=1.15 p=0.887  Complex 13: χ2=1.82 p=0.767 | Simple 9: U=1068 p=0.414  Complex 9: U=908 p=0.948  Complex 13: U=1068 p=0.414 | Simple 9: ρ=0.084 p=0.435  Complex 9: ρ=-0.121 p=0.268  Complex 13: ρ=0.012 p=0.911 | Simple 9: ρ=0.160 p=0.135  **Complex 9: ρ=-0.180 p=0.097**  Complex 13: ρ=-0.074 p=0.492 |
| Face pop-out face peak look duration | ρ=-0.171 p=0.153 | χ2=5.43 p=0.246 | **U=800 p=0.019** | ρ=-0.023 p=0.850 | ρ=-0.020 p=0.869 |
| Results in bold (p<0.1) were entered as covariates in all analyses | | | | | |

| Table S2. Associations between potential covariates and effortful control scores | | | |
| --- | --- | --- | --- |
| Task | Corrected Age at Assessment | Multiple Deprivation Index Quintile | Sex |
| Gap-overlap | ρ=0.016 p=0.887 | H=0.516 p=0.972 | U=702 p=0.328 |
| Cognitive control- reversal learning | ρ=-0.125 p=0.362 | H=2.17 p=0.704 | **-** |
| Visual search | **-** | H=1.21 p=0.877 | U=814 p=0.422 |
| Face pop-out | ρ=0.012 p=0.923 | H=2.20 p=0.699 | **-** |

| Table S3. F-statistics and p-values for all covariates included in ANCOVAs examining differences between toddlers with CHD and controls | | | | | | | | |
| --- | --- | --- | --- | --- | --- | --- | --- | --- |
|  | | Gestational age at birth | Cognitive composite score | Sex | Accuracy | Degree | Corrected age at assessment | Condition |
| Gap-overlap | Reaction times | F(1,75)=3.22 p=0.077 | F(1,75)=0.122 p=0.728 | F(1,75)=2.53 p=0.116 | F(1,75)=0.453 p=0.116 | F(1,75)=0.681 p=0.412 | - | F(1,160)=282 p<0.001 |
| Non-social contingency | reaction times to pick a ball | F(1,52)=4.13 p=0.047 | F(1,52)= 0.247 p=0.621 | F(1,52)=0.644 p=0.426 | - | - | F(1,52)=0.260 p=0.612 | F(1,112)=1.26 p=0.287 |
|  | reaction times to fixation | F(1,50)=1.33 p=0.255 | F(1,50)=1.51 p=0.225 | F(1,50)=0.468 p=0.497 | F(1,50)=1.90 p=0.174 | F(1,50)=2.26 p=0.139 | F(1,50)=0.102 p=0.751 | F(1,112)=7.68 p<0.001 |
| Cognitive control- reversal learning | Proportion of correct saccades | F(1,51)=2.30 p=0.136 | F(1,51)=2.72 p=0.105 | F(1,51)=2.53 p=0.117 | - | - | - | F(1,54)=06793 p=0.414 |
|  | Reaction times | F(1,52)=0.900 p=0.347 | F(1,52)=0.036 p=0.851 | - | - | - | - | F(1,54)=0.433 p=0.514 |
| Visual Search | Proportion of correct trials | F(1,81)=0.2.15 p=0.147 | F(1,81)=0.047 p=0.829 | F(1,81)=2.00 p=0.161 | F(1,81)=1.09 p=0.299 | F(1,81)=3.37 p=0.040 | - | F(1,172)=56.2 p<0.001 |
|  | Reaction times | F(1,79)=0.520 p=0.473 | F(1,79)=0.064 p=0.801 | - | F(1,79)=0.090 p=0.764 | - | F(1,79)=0.772 p=0.418 | F(1,166)=56.2 p<0.001 |
| Face pop-out | Peak look duration at faces | F(1,65)=1.67 p=0.200 | F(1,65)=0.014 p=0.904 | F(1,65)=0.945 p=0.335 | - | - |  | - |

| Table S4. Regression coefficients and p-values for all covariates included in regression models predicting eye-tracking measures from effortful control*CHD | | | | | | | | |
| --- | --- | --- | --- | --- | --- | --- | --- | --- |
|  | | Gestational age at birth | Cognitive composite score | Sex | Accuracy | Degree | Corrected age at assessment | Intercept |
| Gap-overlap | Baseline reaction times | B(SE)=-0.22 (0.009) p=0.019 | B(SE)<0.001 (0.001) p=0.947 | - | B(SE)=-0.017 (0.034) p=0.628 | B(SE)=0.012 (0.058) p=0.832 | - | B(SE)=6.71 (0.431) p<0.001 |
|  | Gap reaction times | B(SE)=-0.01 (0.008) p=0.237 | B(SE)= 0.003 (0.001) p=0.040 | - | B(SE)=0.013 (0.030) p=0.654 | B(SE)=0.094 (0.051) p=0.069 | - | B(SE)=5.62 (0.386) p<0.001 |
| Cognitive control- reversal learning | Proportion of correct saccades | B(SE)=-0.062 (0.051) p=0.232 | B(SE)= 0.010 (0.008) p=0.205 | B(SE)=0.129 (0.159) p=0.417 | - | - | - | B(SE)=0.333 (2.83) p=0.906 |
| Visual Search | Simple 9 reaction time | B(SE)=29.1 (16.6) p=0.084 | B(SE)=0.333 (2.74) p=0.904 | - | B(SE)=35.5 (35.3) p=0.317 | - | B(SE)=-15.8 (8.26) p=0.059 | B(SE)=171.6 (729.8) p=0.829 |
|  | Complex 9 reaction time | B(SE)=-12.7 (28.8) p=0.152 | B(SE)=-1.17 (4.69) p=0.804 | - | B(SE)=-64.4 (60.8) p=0.293 | - | B(SE)=-12.8 (14.2) p=0.369 | B(SE)= 1979 (1367) p=0.152 |
|  | Complex 13 reaction time | B(SE)=25.6 (34.6) p=0.461 | B(SE)= -2.19 (5.67) p=0.700 | - | B(SE)=4.29 (73.4) p=0.954 | - | B(SE)=16.1 (17.2) p=0.350 | B(SE)= -269 (1642) p=0.870 |
| Face pop-out | Peak look duration at faces | B(SE)=-0.025 (0.019) p=0.196 | B(SE)<0.001 (0.003) p=0.861 | B(SE)=-0.049 (0.063) p=0.439 | - | - |  | - |

| Table S5. Eye-tracking data quality in toddlers in CHD | | | |
| --- | --- | --- | --- |
|  | CHD | Control | Difference |
| Accuracy, median (IQR) | 1.5 (1.3-1.9) | 1.6 (1.3-1.9) | U=1013, p=0.859 |
| Precision, median (IQR) | 1.5 (1.2-1.8) | 1.5 (1.3-1.8) | U=1017, p=0.834 |
| Gap-overlap valid data, n (%) | 25 (83) | 58 (88) | p=0.537 |
| Pop out valid data, n (%) | 22 (73) | 49 (74) | χ2=0 p=1.00 |
| Visual Search valid data, n (%) | 28 (93) | 61 (92) | p=1.00 |
| Cognitive Control- reversal learning valid data, n (%) | 22 (73) | 38 (58) | χ2=1.56 p=0.211 |
| Non-social contingency valid data, n (%) | 21 (70) | 38 (58) | χ2=0.871 p=0.351 |

| Table S6. Relationships between effortful control and eye-tracking metrics across groups | |
| --- | --- |
| Gap-overlap Baseline mean reaction time | B(SE)=-0.030 (0.020), p=0.140 |
| Gap-overlap Gap mean reaction time* | B(SE)=-0.024 (0.019), p=0.206 |
| Cognitive control- reversal learning reversal condition proportion correct^ | B(SE)=0.073 (0.117) p=0.535 |
| Visual search simple 9 mean reaction time* | B(SE)=-144 (88) p=0.147 |
| Visual search complex 9 mean reaction time* | B(SE)=5.84 (54.6) p=0.915 |
| Visual search complex 13 mean reaction time | B(SE)=12.7 (184) p=0.873 |
| Pop-out face peak look duration | B(SE)=-4.20 (63.6) p=0.913 |
| Results in bold are significant  *robust regression  ^poisson regression | |
